# Comparing two identically protocolized, multicentre, randomized controlled trials on caregiver-mediated exercises poststroke: Any differences across countries?

**DOI:** 10.1371/journal.pone.0263013

**Published:** 2022-01-25

**Authors:** Marijn Mulder, Rinske H. M. Nijland, Judith D. M. Vloothuis, Maayken van den Berg, Maria Crotty, Gert Kwakkel, Erwin E. H. van Wegen

**Affiliations:** 1 Department of Rehabilitation Medicine, Amsterdam Movement Sciences, Amsterdam UMC, VU University Medical Centre, Amsterdam, The Netherlands; 2 Rehabilitation Research Centre, Reade, Amsterdam, The Netherlands; 3 Department of Rehabilitation, Aged and Extended Care, Flinders University, Adelaide, Australia; 4 Amsterdam Neuroscience, Amsterdam, The Netherlands; 5 Department of Physical Therapy and Human Movement Sciences, Northwestern University, Chicago, Illinois, United States of America; University of Kansas Medical Center, UNITED STATES

## Abstract

**Background:**

The evidence for rehabilitation interventions poststroke lack sufficient robustness. However, variation in treatment effects across countries have been given little attention.

**Objective:**

To compare two identically protocolized trials conducted in different western countries in order to identify factors that may have caused variation in secondary trial outcomes.

**Methods:**

Comparative study based on individual patient data (N = 129) from two randomized controlled trials, conducted in hospitals and rehabilitation facilities in the Netherlands (N = 66) and Australia (N = 63). Patients with stroke and their caregivers were randomly allocated to an 8-week caregiver-mediated exercises intervention (N = 63; 31 Australian and 32 Dutch) or to a control group (N = 66; 32 Australian and 34 Dutch). Patient characteristics, compliance, usual care and process measures were compared across countries. We examined if study setting significantly moderated the trial outcomes: Hospital Anxiety and Depression Scale, Fatigue Severity Scale and General Self-Efficacy Scale, measured at 8- and 12 weeks follow-up. In addition, we explored if factors that were significantly different across countries caused variation in these trial outcomes.

**Results:**

Most patients suffered an ischemic stroke, were in the subacute phase and participated with their partner. Dutch patients were younger (*P* = 0.005) and had a lower functional status (*P* = 0.001). Australian patients were recruited earlier poststroke (*P*<0.001), spent less time in exercise therapy (*P*<0.001) and had a shorter length of stay (*P*<0.001). The level of contamination was higher (*P* = 0.040) among Dutch controls. No effect modification was observed and trial outcomes did not change after controlling for cross-country differences.

**Conclusions:**

The present study highlighted important clinical differences across countries whilst using an identical study protocol. The observed differences could result in a different potential for recovery and variation in treatment effects across trials. We argue that we can proceed faster to evaluating interventions within international pragmatic trials.

## Introduction

Stroke affects approximately 104 million people worldwide and the global incidence of stroke is currently estimated at 11.9 million [1]. Stroke is a disabling condition with variable recovery patterns and a heterogeneous functional outcome [2]. Most patients require rehabilitation to assist functional recovery and optimize independence in daily life [2, 3]. However, the current evidence for specific rehabilitation interventions poststroke lacks sufficient robustness and is largely based on findings from small, phase II, Randomized Controlled Trials (RCTs) [3–5]. Phase II RCTs are designed to estimate treatment effects with the lowest possible bias. Therefore, conflicting results across trials is assumed to result from either imprecision of effect estimates or true effect variation as a result of clinical heterogeneity [6]. Clinical heterogeneity refers to systematic differences in the outcomes studied or timing of outcome measurement, characteristics of patients (e.g. age, ethnicity, disease severity), interventions (e.g. content, dose, duration), or study setting (e.g. country, healthcare system, cultural context) [7]. Further investigation of responder and non-responder groups or dose-response relationships is common within stroke recovery trials. However, variation in treatment effects across countries with a different healthcare system and cultural context have been given little attention.

In a recent collaboration, we simultaneously conducted two RCT’s in Australia (ACTRN12613000779774) and the Netherlands (NTR4300). Both studies investigated the effect of a Caregiver-Mediated Exercises (CME) intervention with e-health support [8, 9], which may be a valuable intervention to augment exercise therapy and improve functional outcome after stroke [10, 11]. Although the primary outcome of both trials was neutral with respect to patients’ self-reported mobility, different significant results regarding secondary psychosocial outcomes were found. The Dutch trial reported a significant decrease in caregiver depression and patient anxiety [9], while the Australian trial reported a significant decrease in caregiver fatigue and increased caregiver self-efficacy [8]. Both trials had an identical study design that obeys CONSORT statements, used the same measurements of outcome and identical criteria of patient selection [12]. In addition, an identical intervention protocol in terms of content, dosing and treatment goals was used [13]. However, the studies were embedded within a different healthcare system and cultural context, making study setting the main source of heterogeneity for possible differences in outcome. Combining individual patient data from both trials allows us to investigate clinical heterogeneity and true effect variation across countries providing valuable information for future trial design and conduct.

The aim of the current study was to compare two identically protocolized trials in terms of design, outcome measurement, criteria of patient selection, and intervention protocol, applied in the Netherlands and Australia in order to identify factors that may have caused variation across countries in secondary trial outcomes. First, we hypothesized that the identical criteria of patient selection reduced patient heterogeneity. Second, we hypothesized that significant differences exist across countries in terms of usual exercise therapy and processes of care including length of inpatient stay and timing of recruitment. Finally, we hypothesized that study setting significantly moderated the secondary trial outcomes anxiety, depression, fatigue and self-efficacy.

## Material and methods

### Study design

This study combined individual patient data from two identically protocolized, observer-blinded, multicentre RCTs in which patients with stroke and their appointed caregivers were randomly allocated to a CME intervention with e-health support in addition to usual care or to a control group that received usual care alone [12]. The two trials were simultaneously started in the Netherlands and Australia, respectively. The randomization procedures, sample size calculations and results of the separate trials have been published previously [8, 9]. The medical ethics committee of the Slotervaart Hospital and Reade approved the Dutch study (NTR4300) and the local health research ethics board of Southern Adelaide Research Ethics Committee approved the Australian study (ACTRN12613000779774).

### Definitions

Imprecision is defined as random error in trial outcomes, as a result of sample variation. Hence, precision of effect estimates depends on the sample size, with smaller studies being less precise [6].

Methodological heterogeneity is defined as a difference across trials in the study design, outcome measurement tools or risk of bias. A higher degree of bias will lead to systematic error in trial outcomes [6].

Clinical heterogeneity is defined as a systematic difference across trials in the outcomes studied or timing of outcome measurement, characteristics of patients (e.g. age, ethnicity, disease severity), interventions (e.g. content, dose, duration), or study setting (e.g. country, healthcare system, cultural context) [6, 7].

True effect variation is defined as variation in treatment effects as a consequence of clinical heterogeneity. True variation in trial outcomes is not explained by random or systematic error [6, 7].

Study protocol is defined as a combination of the study design and intervention protocol. The design characteristics (e.g., blinding, randomization), study procedures, outcome measures, power calculation, criteria of patient selection and statistical analysis are protocolized in the study design. The essential elements of the trial intervention including its content, mode of delivery, dose, duration and monitoring of compliance are protocolized in the intervention protocol.

Study setting is defined as the geographical location of a trial (i.e., country) including the healthcare system and cultural context in which it is embedded, and the context of individual study sites including their level of care (i.e., primary, secondary, tertiary), local healthcare processes and available resources including staff and expertise [14].

Contamination is defined as the receipt of active intervention amongst participants in the control arm of an RCT [15].

Pragmatic trials are defined as RCTs designed to determine the effect of an intervention under the usual conditions in which it will be applied [16], more specifically the trial should 1) enrol a real-world population; 2) be conducted in a real-world setting; 3) include an appropriate comparison arm; and 3) capture relevant outcomes [17].

### Participant recruitment

Patients in the Netherlands were recruited from four hospital stroke units, two rehabilitation centres and seven rehabilitation wards of nursing homes in Amsterdam and its near surroundings. Patients in Australia were recruited from two hospital stroke units and one hospital rehabilitation unit in metropolitan Adelaide. Eligibility criteria for both patients and caregivers were: 1) 18 years or older; 2) able to understand the Dutch or English language; and 3) no significant signs of depression (Hospital Anxiety and Depression Scale [HADS] depression subscale < 11). Patients were eligible if they: 1) were diagnosed with stroke according to the WHO definition [18]; 2) experienced mobility limitations (Functional Ambulation Categories score < 5); 3) had sufficient cognition to follow instructions and provide informed consent (Mini-Mental State Examination [MMSE] > 18); 4) lived independently prior to stroke; 5) were planned to be discharged home; and 6) were willing and able to appoint a caregiver. Caregivers were eligible if they were: 1) willing to participate in CME; and 2) physically able to support the patient. Exclusion criteria for both patients and caregivers were: 1) a serious comorbidity that interfered with participation; and 2) not being medically stable. Participants were recruited in the early rehabilitation phase, following admission in a participating centre. All participants provided written informed consent prior to study enrolment.

### Intervention

The C4S program consisted of an 8-week incremental training program with task-oriented and mobility-related exercises. An identical treatment protocol, including the intended dose, following the TIDieR guidelines was used [13]. Patient-caregiver dyads were asked to perform exercises together, at least five times a week for 30 minutes, within the clinical setting, outside or at home. In addition, participants were encouraged to exercise outside usual training hours. During a weekly face-to-face session with a trained physical therapist, dyads were instructed, evaluated and caregivers were educated to become an exercise coach. During the program, dyads were supported by an offline e-health application with instruction videos, in addition to usual care. The Australian and Dutch trials used respectively an English and Dutch version of the same video application.

### Primary and secondary outcomes

Patients and caregivers in both trials were assessed using the same outcome measures, at baseline prior to randomization, and at 8- and 12-weeks follow-up. In both countries, outcome assessments were completed by an independent assessor blinded to treatment allocation. The primary outcome measure of both trials was the mobility domain of the Stroke Impact Scale (SIS 3.0) [19]. The secondary outcome measures including their description and references has been published previously [12]. For the purpose of the current investigation, we selected the anxiety and depression subscales of the HADS [20], the Fatigue Severity Scale (FSS) [21] and General Self-Efficacy Scale (GSES) [22] of both patients and caregivers to assess secondary trial outcomes of anxiety, depression, fatigue and self-efficacy.

To investigate systematic differences across countries in patient characteristics we used sex, age, living situation prior to stroke (alone or with someone), relationship with the appointed caregiver, type of stroke (ischemic or haemorrhagic), site of stroke (left or right hemisphere), functional status measured with the modified Rankin Scale (mRS) [23] and cognition measured with the MMSE [24] at the moment of recruitment. Self-reported exercise minutes from diaries that were kept for 8 weeks following randomization, were used to investigate systematic differences in compliance and contamination (CME minutes) and usual exercise therapy (exercise minutes with a nurse, therapist and independent exercise minutes). Process measures that were compared across countries included timing of recruitment (days between stroke onset and randomization), timing of discharge (days between randomization and inpatient discharge) and Length Of inpatient Stay (LOS) which was defined as the number of days between hospital admission and discharge from inpatient care.

### Statistical analyses

Individual patient data from both trials were merged in one pooled database. First, systematic differences across the Dutch and Australian trials in terms of 1) patient characteristics; 2) compliance and usual exercise therapy; and 3) process measures; were compared using independent samples t-tests and we reported means and standard deviations in case of a normal distribution or used Mann-Whitney U tests and reported medians and interquartile ranges in case of a skewed distribution. A Chi-square test was used for categorical outcomes or a Fisher’s exact test in case of few observations for individual cells (<10). Second, Linear Mixed Models were used to examine if the dichotomous variable ‘country’ was an effect-modifier that significantly moderated the secondary trial outcomes, with HADS anxiety, HADS depression, FSS and GSES as the dependent variables and treatment allocation, country, baseline score of the dependent variable and a treatment-by-country interaction term as covariates. A random intercept was included to adjust for the dependency of the repeated observations within subjects [25]. If a significant interaction was detected, treatment effects were reported separately for both countries. If no significant interaction was identified, the overall treatment effect was reported while controlling for country. Finally, patient characteristics and process measures that were systematically different across countries were successively added to the longitudinal model in order to explore if these factors may have caused variation in secondary trial outcomes. If the regression coefficient of treatment allocation changed by ≥10%, the covariate was considered to be a confounder and new candidate confounders were added to the longitudinal model [26, 27]. A 2-tailed significance level alpha of 0.05 was used for all the statistical tests that were performed. Data were analysed using IBM SPSS Statistics version 25 for Windows.

## Results

Individual patient data of 129 patients and their appointed caregivers were included, of which 66 dyads were recruited in the Netherlands and 63 dyads in Australia. A total of 617/709 screened (87.0%) in Australia and 960/1082 screened (88.7%) in the Netherlands were excluded from participation. Patients were selected according to the same eligibility criteria, and no additional strategies were used to select or exclude patients. No significant difference (*P* = 0.054) was observed in the proportion of eligible patient-caregiver dyads that declined to participate in Australia, 29/709 screened (4.1%), compared to those that declined to participate in the Netherlands, 56/1082 screened (5.2%).

### Systematic differences in patient characteristics

Table 1 presents the baseline characteristics of recruited patients in both countries. Patients in the Dutch trial were significantly younger (MD 7.7 years, CI 2.3–13.0; *P* = 0.005) and had a lower functional level (82% with a moderate to severe disability versus 54% in Australia) when compared to Australian patients. Although a higher proportion of patients were living alone prior to stroke in the Netherlands, this difference did not reach significance.

**Table 1:** Differences in patient characteristics and process measures.

Patient characteristics	The Netherlands (n = 66)	Australia (n = 63)	P-value
Age in years, mean (SD)	59.9 (14.8)	67.5 (15.8)	0.005* ^a^
Sex, number of males (%)	41 (62.1)	40 (63.5)	0.872^c^
Type of stroke, N (%)			0.115^d^
Ischemic	50 (75.8)	54 (85.7)	
Haemorrhagic	16 (24.2)	8 (12.7)	
Both	0 (0.0)	1 (1.6)	
Site of stroke, N (%)			0.592^d^
Right hemisphere	37 (56.1)	39 (61.9)	
Left hemisphere	28 (42.4)	24 (38.1)	
Brainstem	1 (1.5)	0 (0.0)	
mRS disability (0–5), N (%)			0.001* ^d^
1 no significant	1 (1.5)	2 (3.2)	
2–3 slight-moderate	11 (16.7)	27 (42.9)	
4–5 moderate-severe	54 (81.8)	34 (53.9)	
MMSE (0–30), median (IQR)	28.0 (25.0–29.0)	26.0 (24.0–28.3)	0.226^b^
Living alone, N (%)	20 (30.3)	10 (15.9)	0.059^c^
Caregiver, N (%)			0.280^c^
Partner	39 (59.1)	43 (68.3)	
Another caregiver	27 (40.9)	20 (31.7)	
Timing of recruitment in days, median (IQR)	37 (28–56)	14 (7–22)	<0.001* ^b^
Timing of discharge in days, median (IQR)	69 (41–92)	18 (7–35)	<0.001* ^b^
LOS in days, median (IQR)	112 (79–148)	34 (18–56)	<0.001* ^b^

IQR, inter quartile range; SD, standard deviation; mRS, modified Rankin Scale; MMSE, Mini-Mental State Examination; LOS, length of inpatient stay.

^a^ *p* values were calculated using independent samples t-tests,

^b^ Mann-Whitney U tests,

^c^ Chi-square tests,

^d^ Fisher’s exact tests

### Systematic differences in compliance and usual care

Compliance with the intervention protocol did not differ across Australian (N = 31, Median 953 CME minutes, IQR 635–1390) and Dutch (N = 32, Median 1150 CME minutes, IQR 850–1500) intervention groups (P = 0.383). However, Australian controls reported significantly fewer CME minutes (N = 32, Median 60 minutes, IQR 5–495; P = 0.040) than Dutch controls (N = 34, Median 350 minutes; IQR 95–1065). Patients in Australia reported significantly (P = 0.04) less usual exercise minutes during the 8 weeks following randomization (N = 63, Median 2550 minutes, IQR 1478–3683) when compared to the Netherlands (N = 66, Median 3403 minutes, IQR 2220–3900). More specifically, patients in Australia reported less minutes in formal exercise therapy (Median 1080 minutes, IQR 473–1735; versus Median 2025 minutes, IQR 1368–2858; P<0.001), but more independent exercises (Median 930 minutes, IQR 300–2408; versus Median 550 minutes, IQR 190–1200; P<0.037). We found no significant difference (P = 0.114) in exercise time with a nurse (Median 0 minutes, IQR 0–140 and Median 35 minutes, IQR 0–173, in Australia and the Netherlands respectively).

### Systematic differences in process measures

In Table 1 process measures are presented for both countries. All process measures differed significantly across countries (*P*<0.001). Patient-caregiver dyads in the Australian trial were recruited significantly earlier poststroke when compared to the Netherlands. Moreover, LOS was significantly shorter in Australia and as a result discharge occurred earlier in the program. Australian patients generally returned home in the first five weeks following randomization while Dutch patients returned home in the last two weeks or after completing the 8-week program.

### Differences in secondary trial outcomes across countries

No significant interaction effects were observed between treatment allocation and the variable ‘country’ with respect to anxiety, depression, self-efficacy and fatigue of both patients and caregivers. The overall treatment effects of the C4S program with country as a covariate, and the interaction effects of treatment allocation and country are summarized in Table 2. The observed trial outcomes did not change after controlling for age, baseline functional status, or after controlling for usual exercise dose, timing of recruitment, timing of discharge and LOS.

**Table 2:** Secondary treatment effects and study setting interaction effects.

**Outcome measures patient**	**Beta (95% CI)**	**P-value** ^a^ **Treatment**	**P-value** ^a^ Treatment*Country
HADS anxiety (0–21)	1.10 (0.01–2.20)	0.049*	0.220
HADS depression (0–21)	0.66 (-0.40–1.72)	0.222	0.470
FSS (1–7)	0.19 (-0.31–0.68)	0.462	0.505
GSES (10–40)	-0.42 (-1.99–1.14)	0.593	0.942
**Outcome measures caregiver**	**Beta (95% CI)**	**P-value** ^a^ **Treatment**	**P-value** ^a^ Treatment*Country
HADS anxiety (0–21)	0.23 (-0.94–1.41)	0.692	0.541
HADS depression (0–21)	0.94 (0.00–1.88)	0.050*	0.412
FSS (1–7)	0.18 (-0.19–0.54)	0.341	0.990
GSES (10–40)	-0.44 (-1.71–0.83)	0.493	0.703

HADS, Hospital Anxiety and Depression Scale; FSS, Fatigue Severity Scale; GSES, General Self-Efficacy Scale.

^a^ *p* values were calculated using Linear Mixed Model analyses

## Discussion

To our knowledge, this is the first study in stroke rehabilitation that investigates cross-cultural clinical differences by comparing two identically protocolized phase II trials with respect to design, outcome measurement, patient selection and intervention conducted in two different Western countries. The present study shows that, despite identical criteria of patient selection, systematic clinical differences may be observed in study participants across countries. In addition, heterogeneity was observed in the level of contamination, amount of usual care and total length of inpatient stay. Awareness of clinical heterogeneity across countries is especially important when designing and conducting global trials. However, study setting did not moderate the secondary trial outcomes anxiety, depression, fatigue and self-efficacy suggesting that we can proceed faster to evaluating interventions within heterogeneous populations and study settings in pragmatic phase III and IV trials.

Patient heterogeneity was observed across countries with older age and a higher functional level at baseline in Australia when compared to the Netherlands. In general, age and functional status after stroke are factors known to influence discharge disposition and referral to inpatient rehabilitation after acute care on a Hospital Stroke Unit (HSU) [28–30]. In the Dutch study, participating HSUs were unable to recruit patients as a result of their fast discharge policies with a short LOS in the hospital. Hence, all patients were recruited from rehabilitation centres and rehabilitation wards of nursing homes. In contrast, Australian patients were recruited from acute HSUs as well as from Hospital Rehabilitation Units which may explain the different casemix. In parallel, Australian patients were recruited earlier poststroke which is not unexpected within an acute setting. Age and functional status at admission are significant predictors of functional outcome after stroke [28, 31]. Moreover, functional improvement is significantly associated with progress of time [32]. This suggests that age, functional status and time poststroke at recruitment may be important effect modifiers that can cause variation in functional outcomes across trials despite following an identical study protocol.

The present study also shows that usual care is strongly dependent on features of the healthcare system and cultural context. To our knowledge, there are no recent studies reporting about practice variation in usual care across countries. We show that Australian patients spent less time in formal exercise therapy in both groups and total LOS was shorter when compared to the Netherlands. For the purpose of this study, LOS was defined as the number of days between acute hospital admission and final discharge home. Hence, this finding is not explained by timing of recruitment or level of care. The shorter LOS in Australia may be explained by the less severely affected population [33, 34] or reflect differences in local healthcare policies with respect to rehabilitation services such as Early Supported Discharge (ESD) [35]. These findings are supported by lower costs of care observed in Australia, when compared to the Netherlands [36]. Although the emphasis on ESD has accelerated the interest in outpatient services [37], most resources in stroke rehabilitation are focused on inpatient care [35, 38]. As a result of the shorter LOS, Australian patients returned home faster following randomization which could explain the lower dose of formal exercise therapy during the 8 weeks thereafter. In stroke rehabilitation trials, the control group often receives ‘usual care’. Unfortunately, the content, timing and dose of usual care is often poorly described [39]. Acknowledging that a difference in the amount of usual care delivered can result in a different potential for recovery [40, 41] it is important to realize that these differences may exist across countries when designing global trials.

In the two trials, it was intended that controls received only usual care [12, 13]. Although some contamination was present in the control groups of both studies, a significant difference was observed in its magnitude. The self-reported dose of CME within control groups was about 8 weekly minutes among Australian controls and 44 weekly minutes among Dutch controls, resulting in a larger treatment contrast in Australia. One of the most common sources of contamination within clinical trials is communication between trial arms, either between staff members or participants [15]. Such communication is more likely within an inpatient environment where patients are admitted to the same rehabilitation ward and providers of the intervention work closely together with other healthcare professionals. The earlier timing of discharge in Australia may therefore partly explain the lower level of contamination in this study. Another common source of contamination is when the intervention is already part of usual care to some extent [15]. Although the involvement of caregivers may differ across different social, cultural, political and organizational contexts [10], the total level of contamination is low and we have no data to support the structural use of CME in a usual care context in the Netherlands.

The present study highlighted important clinical differences across countries whilst using an identical study protocol. Although different significant results were reported in the separate trials regarding secondary outcomes of anxiety, depression, self-efficacy and fatigue [8, 9], we did not find a moderating effect of study setting on these trial outcomes. This suggests that treatment effects on secondary outcomes within small phase II trials may be imprecise and should be interpreted with caution. We argue that we should proceed faster to evaluating promising rehabilitation interventions poststroke in large phase III and IV trials. International collaboration to standardize study protocols including study design, outcome measurement and reporting is an important next step to improve robustness of trial outcomes in the field of stroke rehabilitation [42–44]. Awareness of clinical heterogeneity and consensus in trial conduct across countries through the international GAINS network is especially important when designing future global trials. First, we recommend that patients are recruited based on broad selection criteria, however at fixed times or within a narrow time window poststroke, and from multiple study settings to improve generalizability. Subsequently, responder analyses can be used to investigate and interpret a heterogeneous response to treatment. Second, the control condition should be monitored during the study period and we should improve our reporting of ‘usual care’ including its specific content, dose and location. Third, careful consideration of the processes driving contamination is needed. Although cluster randomization is often mentioned as a solution, it could lead to patient heterogeneity across trial arms when patients are recruited from different study settings [45]. A balanced distribution of clusters over different settings or changes in trial conduct to reduce contamination could be considered. Finally, international consensus on parameters of trial conduct and reporting is essential for improving the quality of future stroke rehabilitation trials.

### Strengths and limitations

Our individual patient dataset from two identically protocolized trials, independently conducted in two countries with a different healthcare system and cultural context, is unique. However, several study limitations should be acknowledged. First, the current study investigated differences across two Western, high-income countries and no generalizations can therefore be made to non-Western, low- or middle-income countries. Second, we did not collect information on ethnicity, racial groups or comorbidities. Third, timing of recruitment was standardized based on the moment of admission to a participating rehabilitation ward. As a result, randomization, outcome measurement and initiation of the C4S program occurred at relatively arbitrary time points poststroke.

### Conclusion

The present study highlighted important clinical differences across countries whilst using an identical study protocol, providing valuable information for future trial design and conduct. Theoretically, the observed clinical differences could result in a different potential for recovery and variation in treatment effects across trials. However, we did not find a moderating effect of study setting on the current secondary trial outcomes. We argue that we can proceed faster to evaluating promising interventions within large, pragmatic trials and give recommendations for designing future stroke rehabilitation trials.

## Supporting information

S1 Dataset (SAV)
